# Cost‐effectiveness of first‐line immunotherapies for advanced non‐small cell lung cancer

**DOI:** 10.1002/cam4.5632

**Published:** 2023-01-18

**Authors:** Szu‐Chun Yang, Huang‐Tz Ou, Wu‐Chou Su, Shi‐Yi Wang

**Affiliations:** ^1^ Department of Internal Medicine, National Cheng Kung University Hospital, College of Medicine National Cheng Kung University Tainan Taiwan; ^2^ Institute of Clinical Pharmacy and Pharmaceutical Sciences, College of Medicine National Cheng Kung University Tainan Taiwan; ^3^ Department of Pharmacy, College of Medicine National Cheng Kung University Tainan Taiwan; ^4^ Department of Oncology, National Cheng Kung University Hospital, College of Medicine National Cheng Kung University Tainan Taiwan; ^5^ Department of Chronic Disease Epidemiology Yale University School of Public Health New Haven Connecticut USA; ^6^ Cancer Outcomes, Public Policy, and Effectiveness Research (COPPER) Center Yale University School of Medicine New Haven Connecticut USA

**Keywords:** atezolizumab, cost‐effectiveness, immunotherapy, lung cancer, nivolumab, pembrolizumab

## Abstract

**Background:**

Researchers have not simultaneously compared the cost‐effectiveness of six immunotherapies with chemotherapy for advanced non‐small cell lung cancer. This study evaluated the cost‐effectiveness across different programmed death‐ligand 1 (PD‐L1) levels.

**Methods:**

A Markov model with lifetime horizon was created for seven regimens: pembrolizumab plus chemotherapy (pembro‐chemo), nivolumab plus ipilimumab (nivo‐ipi), nivolumab, ipilimumab plus chemotherapy (nivo‐ipi‐chemo), atezolizumab plus chemotherapy (atezo‐chemo), atezolizumab, bevacizumab plus chemotherapy (atezo‐beva‐chemo), single‐agent pembrolizumab, and chemotherapy alone. Input parameters were derived from trial data, a network meta‐analysis, and other literature. We conducted the analysis from the perspective of US health care sector.

**Results:**

For all patients without considering PD‐L1 expression, the incremental cost‐effectiveness ratio (ICER) of pembro‐chemo versus chemotherapy was $183,299 per quality‐adjusted life year (QALY). The preferred regimens based on ICERs differed by PD‐L1 levels. For patients with PD‐L1 ≥50%, pembrolizumab versus chemotherapy and pembro‐chemo versus pembrolizumab resulted in ICERs of $96,189 and $198,913 per QALY, respectively. The other strategies were dominated. For patients with PD‐L1 of 1%–49%, the ICER of pembro‐chemo comparing to chemotherapy was $218,159 per QALY. The other regimens were dominated by pembro‐chemo. For patients with PD‐L1 <1%, nivo‐ipi versus chemotherapy and nivo‐ipi‐chemo versus nivo‐ipi resulted in ICERs of $161,277 and $881,975 per QALY, and the other regimens were dominated strategies. At the willingness‐to‐pay threshold of $150,000 per QALY, pembrolizumab had 87% and pembro‐chemo had 1% probabilities being cost‐effective in patients with PD‐L1 ≥50% and 1%–49%, respectively. Nivo‐ipi had a 34% probability being cost‐effective in patients with PD‐L1 <1%.

**Conclusions:**

The PD‐L1 level should be incorporated into treatment decision‐making. Our findings suggest that first‐line pembrolizumab, pembro‐chemo, and nivo‐ipi are the preferred strategies for patients with PD‐L1 ≥50%, 1%–49%, and <1%, respectively.

## Graphical abstract

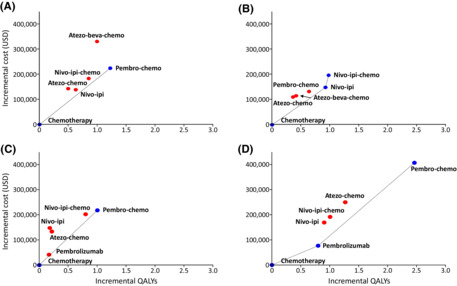

## 1 INTRODUCTION

Lung cancer is the leading cause of cancer death in the world.^1^ About half of non‐small cell lung cancer (NSCLC) patients are diagnosed in advanced stage.^2^ Platinum‐doublet chemotherapy was historically the standard first‐line treatment for patients with advanced NSCLC whose tumors lack of actionable gene alterations. Immunotherapy has changed the landscape of treatments for these patients.^3^

In patients with advanced NSCLC and programmed death‐ligand 1 (PD‐L1) expression levels ≥50% and 1%–49%, first‐line monotherapy with immune checkpoint inhibitor is an effective treatment.^4^, ^5^, ^6^ Immunotherapy in combination with chemotherapy,^7^, ^8^, ^9^, ^10^, ^11^ an anti‐angiogenesis drug,^12^ or another type of immunotherapy^13^, ^14^ can be applied to all patients regardless of PD‐L1 expression levels. Immunotherapy combinations recommended by the National Comprehensive Cancer Network include^15^: pembrolizumab, a programmed death‐1 (PD‐1) antibody, plus chemotherapy; nivolumab, a PD‐1 antibody, plus ipilimumab which blocks cytotoxic T‐lymphocyte antigen 4 (CTLA‐4); nivolumab and ipilimumab plus chemotherapy; atezolizumab, a PD‐L1 antibody, plus chemotherapy; and atezolizumab and chemotherapy plus bevacizumab, which is an anti‐angiogenesis agent. Although all these immunotherapies provide survival benefits for patients with advanced NSCLC, prices tagged on these treatments result in financial pressure on health care system.

Many analyses found that monotherapy with pembrolizumab, as compared to chemotherapy, is a cost‐effective regimen for PD‐L1‐positive NSCLC.^16^, ^17^, ^18^ Studies have also investigated the cost‐effectiveness of pembrolizumab plus chemotherapy,^19^, ^20^, ^21^, ^22^ and nivolumab plus ipilimumab with or without chemotherapy,^23^, ^24^, ^25^, ^26^, ^27^ comparing to platinum‐doublet chemotherapy. However, the results are still inconclusive: Some studies found pembrolizumab plus chemotherapy,^19^, ^22^ and nivolumab plus ipilimumab,^24^ to be cost‐effective; whereas the others did not.^20^, ^21^, ^23^, ^25^, ^26^, ^27^ Literature estimating the incremental cost‐effectiveness ratio (ICER) of atezolizumab plus chemotherapy with or without bevacizumab versus chemotherapy alone has failed to prove the atezolizumab combinations to be cost‐effective.^28^, ^29^, ^30^ More importantly, none of these investigations accounts for three PD‐L1 expression levels (<1%, 1–49%, and ≥ 50%) which are commonly used in practice. Besides, researchers have not simultaneously compared the cost‐effectiveness of these immunotherapies. Based on a network meta‐analysis of these first‐line therapies,^31^ we conducted a cost‐effectiveness analysis evaluating all these immunotherapies across three PD‐L1 expression levels.

## 2 METHODS

### 2.1 Model overview

We created a Markov model to simulate treatment‐naïve advanced NSCLC patients who were treated with one of the seven first‐line therapies^1^: pembrolizumab plus chemotherapy (pembro‐chemo),^2^ nivolumab plus ipilimumab (nivo‐ipi),^3^ nivolumab and ipilimumab plus chemotherapy (nivo‐ipi‐chemo),^4^ atezolizumab plus chemotherapy (atezo‐chemo),^5^ atezolizumab and bevacizumab plus chemotherapy (atezo‐beva‐chemo),^6^ single‐agent pembrolizumab, and^7^ chemotherapy alone (see Figure S1). Because the proportion of deaths attributable to other comorbidities in patients with metastatic NSCLC is minimal,^32^ we assumed no background mortality and all simulated patients entered the model in a progression‐free state and transited to progressive disease before death. We chose a model cycle length of 6 weeks because ipilimumab was administered every 6 weeks,^13^, ^14^ whereas other regimens were administered every 3 weeks.^5^, ^7^, ^8^, ^9^, ^10^, ^11^, ^12^

In accordance with clinical practice, paclitaxel plus carboplatin and pemetrexed plus carboplatin were selected as the baseline chemotherapy regimens for patient with squamous NSCLC and non‐squamous NSCLC, respectively.^15^ Each immunotherapy regimen was allowed to be administered for a maximum of 2 years or up to disease progression. We selected combination chemotherapy according to each trial design. More specifically, platinum‐doublet chemotherapy was administered for 6 weeks in nivo‐ipi‐chemo combination^14^ and 12 weeks in pembro‐chemo,^7^, ^8^ atezo‐chemo,^9^, ^10^, ^11^ and atezo‐beva‐chemo groups.^12^ We considered maintenance therapy with pemetrexed in pembro‐chemo,^7^ atezo‐chemo,^11^ and chemotherapy groups.^15^ Similarly, maintenance therapy with bevacizumab was continued up to disease progression in atezo‐beva‐chemo group.^12^ We modeled the subsequent therapies according to each trial data and standardized the event probabilities based on the chemotherapy group in CheckMate 227 trial.^13^ Because of a lack of such information in the IMpower150 trial,^12^ patients in atezo‐beva‐chemo group were assumed to experience the same subsequent therapies as those in atezo‐chemo group. Docetaxel was used as the second‐line chemotherapy for patients progressed after platinum‐doublet chemotherapy.^15^ We selected nivolumab as the second‐line immunotherapy because it was most popularly used in the trials. Likewise, erlotinib was selected as the second‐line targeted therapy.

### 2.2 Survival estimates

Because the CheckMate 227 trial has the longest follow‐up period and includes both squamous and non‐squamous patients,^13^ we calibrated the progression‐free survival (PFS) and overall survival (OS) to the chemotherapy group and simulated the survival of patients undergoing six different regimens by using the respective hazard ratios of immunotherapies versus chemotherapy from a network meta‐analysis^31^ and the KEYNOTE‐042 study.^5^ There was a lack of PFS and OS curves for patients with PD‐L1 of 1%–49% in the CheckMate 227 trial,^13^ we calibrated the PFS and OS to the chemotherapy group of KEYNOTE‐189 trial.^7^ Hazard ratios of immunotherapies versus chemotherapy^5^, ^31^ were then used to simulate the survival of patients with PD‐L1 of 1%–49% who underwent immunotherapy regimens. A web‐based software (WebPlotDigitizer; https://automeris.io/WebPlotDigitizer/) was applied to extract the data points of PFS and OS curves from the chemotherapy groups. The transitional probability of progression‐free state to progressive disease at each model cycle was directly derived from the PFS curve in the trial, and was time‐dependent. We calibrated the transitional probability of progressive disease to death at each model cycle to fit the OS curve. Based on the transitional probabilities at the end of follow‐up periods, we extrapolated the PFS and OS to lifetime. The modeled PFS and OS curves within the follow‐up periods were compared with the trial results.

The transitional probability at each model cycle of the chemotherapy group was converted to that of each immunotherapy. First, we used the formula, *r* = [−ln (1 − *p*)], to transform probability (*p*) to rate (*r*) at each model cycle. Second, the rate was multiplied by the hazard ratio of immunotherapy versus chemotherapy deriving from the network meta‐analysis^31^ and KEYNOTE‐042 study.^5^ Finally, we used the formula, *p* = 1 − e^−*r*^, to convert the new rate to the transitional probability of each immunotherapy. As such, we were able to simulate the PFS and OS curves of patients receiving six different immunotherapies.

### 2.3 Cost and utility inputs

We considered administration cost, drug costs, costs for best supportive care, and management of adverse events. All these costs were based on the payments by the Centers for Medicare & Medicaid Services.^19^, ^33^, ^34^, ^35^, ^36^, ^37^ We estimated the drug dosages using a body surface area of 1.84 m^2^, a body weight of 70 kg, and a glomerular filtration rate of 73 ml/min (i.e., a 65‐year‐old man with a serum creatinine of 1 mg/ml). We accounted the waste of drugs while calculating costs of intravenous agents (see Table S1). Adverse events considered in the model were those of any grade and we weighted the costs by event rates adjusted in the network meta‐analysis and KEYNOTE‐042 study.^5^, ^31^ Using medical care inflation rates, all costs were made equivalent to 2021 US dollars.

A utility value of 0.79 for patients in the progression‐free state who received chemotherapy alone was obtained from prior research.^38^ A utility ratio denoted the utility value of each immunotherapy divided by the value of its chemotherapy group.^39^, ^40^, ^41^, ^42^, ^43^, ^44^ We multiplied 0.79 by the ratio to derive the adjusted utility value of each immunotherapy. Notably, European Quality of Life Five‐Dimension data were not evaluated in the IMpower trials.^9^, ^10^, ^11^ We alternatively used a mapping approach^45^ by converting the European Organization for Research and Treatment of Cancer Quality of Life‐Core 30 Questionnaire data^42^ to the utility values of patients in atezo‐chemo and atezo‐beva‐chemo groups.^43^ Patients in the progressive disease of seven treatment strategies shared the same utility value of 0.72.^38^

### 2.4 Base‐case analysis

This analysis was conducted from the perspective of US health care sector and we selected a willingness‐to‐pay (WTP) threshold of $150,000 per quality‐adjusted life year (QALY).^46^ We estimated ICERs in terms of incremental costs divided by incremental life years and QALYs, and used an annual rate of 3% to discount future costs and life years. A lifetime horizon and half‐cycle correction were applied. We considered different PD‐L1 expression levels: all patients; patients with PD‐L1 <1%; patients with PD‐L1 of 1%–49%; and patients with PD‐L1 ≥50%. Strategies were rank‐ordered by cost in each group. Strongly dominated strategies were the ones that had higher costs and fewer QALYs than alternative strategies. Weakly dominated strategies were the ones that were less efficient in terms of incremental costs per QALY as compared with alternative strategies. ICERs were calculated against the next costliest and un‐dominated strategy. Amua software version 0.3.0 was used to perform the analyses.

### 2.5 Sensitivity analyses

Our base‐case analysis modeled the PFS and OS of patients undergoing immunotherapies by using the hazard ratios in the network meta‐analysis^31^ and KEYNOTE‐042 study.^5^ Sensitivity analyses using the lower and upper bonds of the 95% confidence intervals of hazard ratios were conducted. Additionally, we performed one‐way deterministic analysis of each group by varying the other parameters within clinically plausible ranges (Table 1 and Table S2). Probabilistic analyses using Monte Carlo simulation with 1000 iterations were done to address the effect of parameter uncertainty. To test the robustness of our results, we compared the base‐case results with results using trial outcomes of immunotherapy combinations.^7^, ^13^

**TABLE 1:** Model parameters^a^

Parameter	Value	Range	Distribution	Source
Squamous in histology	28.0%		Dirichlet (163,419)	CheckMate 227 trial^13^
Chemotherapy, transitional probabilities	Time‐ dependent			Estimated from the PFS and OS curves of CheckMate 227 trial^13^
Pembro‐chemo versus Chemotherapy, HRs				
All, PFS/OS	0.54/0.61	0.49–0.61/0.53–0.70		Network meta‐analysis^31^
PD‐L1 <1%, PFS/OS	0.71/0.71	0.58–0.86/0.57–0.88		Network meta‐analysis^31^
PD‐L1 of 1%–49%, PFS/OS	0.56/0.58	0.45–0.69/0.46–0.73		Network meta‐analysis^31^
PD‐L1 ≥50%, PFS/OS	0.36/0.51	0.29–0.46/0.34–0.68		Network meta‐analysis^31^
Nivo‐ipi versus Chemotherapy, HRs				
All, PFS/OS	0.79/0.73	0.70–0.89/0.65–0.82		Network meta‐analysis^31^
PD‐L1 1%, PFS/OS	0.75/0.62	0.61–0.92/0.51–0.76		Network meta‐analysis^31^
PD‐L1 of 1%–49%, PFS/OS	0.82/0.94	0.71–0.95/0.77–1.14		Network meta‐analysis^31^
PD‐L1 ≥50%, PFS/OS	0.62/0.70	0.51–0.76/0.57–0.86		Network meta‐analysis^31^
Nivo‐ipi‐chemo versus Chemotherapy, HRs				
All, PFS/OS	0.68/0.66	0.58–0.79/0.56–0.77		Network meta‐analysis^31^
PD‐L1 <1%, PFS/OS	0.62/0.62	0.48–0.81/0.48–0.81		Network meta‐analysis^31^
PD‐L1 of 1%–49%, PFS/OS	0.69/0.61	0.51–0.94/0.46–0.80		CheckMate 9LA trial^14^/Network meta‐analysis^31^
PD‐L1 ≥50%, PFS/OS	0.61/0.66	0.42–0.89/0.47–0.92		CheckMate 9LA trial^14^/Network meta‐analysis^31^
Atezo‐chemo versus Chemotherapy, HRs				
All, PFS/OS	0.65/0.83	0.60–0.71/0.75–0.92		Network meta‐analysis^31^
PD‐L1 <1%, PFS/OS	0.70/0.84	0.61–0.79/0.71–0.98		Network meta‐analysis^31^
PD‐L1 of 1%–49%, PFS/OS	0.70/0.95	0.60–0.81/0.77–1.16		Network meta‐analysis^31^
PD‐L1 ≥50%, PFS/OS	0.47/0.64	0.37–0.60/0.47–0.86		Network meta‐analysis^31^
Atezo‐beva‐chemo versus Chemotherapy, HRs				
All, PFS/OS	0.44/0.79	0.36–0.55/0.63–0.99		Network meta‐analysis^31^
PD‐L1 <1%, PFS/OS	0.77/0.79	0.61–0.99/0.57–1.09		IMpower150 trial^12^/Network meta‐analysis^31^
Pembrolizumab versus Chemotherapy, HRs				
PD‐L1 of 1%–49%, PFS/OS	1.03/0.88	0.91–1.16/0.75–1.04		KEYNOTE‐042 trial^5^
PD‐L1 ≥50%, PFS/OS	0.86/0.68	0.72–1.02/0.57–0.81		KEYNOTE‐042 trial^5^
Administration cost, USD	678	543–814	Gamma (100,6.78)	Medicare analysis^19^
Drug cost per 6 weeks, USD				
Pembrolizumab	21,102	16,881‐25,332	Gamma (100,211.02)	Medicare drug prices^33^
Nivolumab	23,090	18,472‐27,708	Gamma (100,230.9)	Medicare drug prices^33^
Ipilimumab	15,865	12,692‐19,038	Gamma (100,158.65)	Medicare drug prices^33^
Atezolizumab	19,140	15,312‐22,968	Gamma (100,191.4)	Medicare drug prices^33^
Bevacizumab	15,491	12,393‐18,589	Gamma (100,154.91)	Medicare drug prices^33^
Pemetrexed	14,986	11,989‐17,983	Gamma (100,149.86)	Medicare drug prices^33^
Carboplatin	63	51–76	Gamma (100,0.63)	Medicare drug prices^33^
Paclitaxel	105	84–126	Gamma (100,1.05)	Medicare drug prices^33^
Docetaxel	134	107–161	Gamma (100,1.34)	Medicare drug prices^33^
Erlotinib	13,147	10,517‐15,776	Gamma (100,131.47)	Medicare analysis^34^
BSC cost per 6 weeks, USD	4574	3659–5488	Gamma (100,45.74)	Medicare analysis^35^
Health utility				
Pembro‐chemo	0.80	0.72–0.88	Beta (19.1,4.7)	EQ‐5D^38^, ^39^
Nivo‐ipi	0.83	0.75–0.92	Beta (15.9,3.2)	EQ‐5D^38^, ^41^
Nivo‐ipi‐chemo	0.81	0.72–0.89	Beta (18.7,4.5)	Time trade‐off^38^, ^40^
Atezo‐chemo	0.80	0.72–0.88	Beta (19.0, 4.7)	Derivation from EORTC^38^, ^42^, ^43^
Atezo‐beva‐chemo	0.80	0.72–0.88	Beta (19.6,5.0)	Derivation from EORTC^38^, ^42^, ^43^
Pembrolizumab	0.84	0.75–0.92	Beta (16.8,3.2)	EQ‐5D^38^, ^44^
Chemotherapy	0.79	0.71–0.87	Beta (20.2,5.4)	EQ‐5D^38^
Progressive disease	0.72	0.65–0.79	Beta (27.3,10.6)	EQ‐5D^38^
Second‐line therapy of Pembro‐chemo				
Chemotherapy	39.8%	31.9%–47.8%	Beta (163,247)	KEYNOTE‐189 trial^7^, ^13^
Immunotherapy	13.4%	10.7%–16.0%	Beta (55,355)	KEYNOTE‐189 trial^7^, ^13^
Targeted therapy	4.6%	3.6%–5.5%	Beta (19,391)	KEYNOTE‐189 trial^7^, ^13^
Second‐line therapy of Nivo‐ipi				
Chemotherapy	35.0%	28.0%–42.0%	Beta (204, 379)	CheckMate 227 trial^13^
Immunotherapy	5.5%	4.4%–6.6%	Beta (32,551)	CheckMate 227 trial^13^
Targeted therapy	5.7%	4.6%–6.8%	Beta (33,550)	CheckMate 227 trial^13^
Second‐line therapy of Nivo‐ipi‐chemo				
Chemotherapy	38.7%	30.9%–46.4%	Beta (140,221)	CheckMate 9LA trial^13^, ^14^
Immunotherapy	7.1%	5.7%–8.5%	Beta (26,335)	CheckMate 9LA trial^13^, ^14^
Targeted therapy	6.4%	5.1%–7.6%	Beta (23,338)	CheckMate 9LA trial^13^, ^14^
Second‐line therapy of Atezo‐chemo				
Chemotherapy	36.2%	29.0%–43.5%	Beta (516,908)	IMpower130,131,132 trials^9^, ^10^, ^11^, ^13^
Immunotherapy	7.2%	5.8%–8.7%	Beta (103,1321)	IMpower130,131,132 trials^9^, ^10^, ^11^, ^13^
Targeted therapy	6.2%	4.9%–7.4%	Beta (88,1336)	IMpower130,131,132 trials^9^, ^10^, ^11^, ^13^
Second‐line therapy of Pembrolizumab				
Chemotherapy	35.2%	28.2%–42.3%	Beta (224,413)	KEYNOTE‐042 trial^5^, ^13^
Immunotherapy	9.5%	7.6%–11.4%	Beta (61,576)	KEYNOTE‐042 trial^5^, ^13^
Targeted therapy	4.8%	3.8%–5.7%	Beta (30,607)	KEYNOTE‐042 trial^5^, ^13^
Second‐line therapy of Chemotherapy				
Chemotherapy	29.7%	23.8%–35.6%	Beta (173,410)	CheckMate 227 trial^13^
Immunotherapy	40.8%	32.6%–49.0%	Beta (238,345)	CheckMate 227 trial^13^
Targeted therapy	5.8%	4.6%–7.0%	Beta (34,549)	CheckMate 227 trial^13^

Abbreviations: BSC, best supportive care; EORTC, European Organization for Research and Treatment of Cancer; EQ‐5D, European Quality of Life Five‐Dimension; HR, hazard ratio; PD‐L1, programmed‐death ligand 1; PFS, progression‐free survival; OS, overall survival; USD, US dollars.

^a^ Parameter values for adverse events are shown in Table S2.

## 3 RESULTS

### 3.1 Base‐case results

**FIGURE 1:**
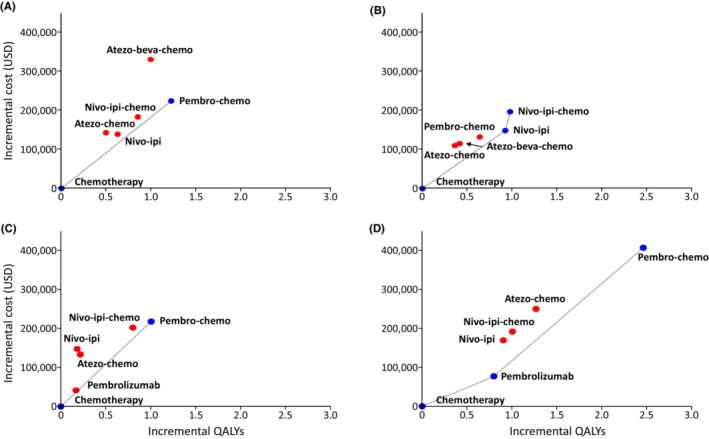
Incremental cost‐effectiveness planes for (A) all patients; (B) patients with PD‐L1 <1%; (C) patients with PD‐L1 of 1%–49%; and (D) patients with PD‐L1 ≥50%. Line‐connected blue dots represent the most efficient strategies. Red dots are dominated strategies. PD‐L1, programmed death‐ligand 1; QALY, quality‐adjusted life year; USD, US dollars.

The modeled PFS and OS curves of chemotherapy group within the follow‐up periods were quite similar to those in the trials (see Figure S2), indicating our model was well calibrated. The results of base‐case analysis and the incremental cost‐effectiveness planes are presented in Table 2 and Figure 1. For all patients without considering PD‐L1 expression, the ICERs of pembro‐chemo versus chemotherapy were $141,790 per life year and $183,299 per QALY. The preferred regimens based on ICERs differed by PD‐L1 expression levels. For patients with PD‐L1 ≥50%, pembrolizumab versus chemotherapy and pembro‐chemo versus pembrolizumab resulted in ICERs of $96,189 and $198,913 per QALY, respectively. The other strategies were dominated. Comparing to chemotherapy, the ICERs of pembro‐chemo were $168,878 per life year and $218,159 per QALY for patients with PD‐L1 of 1%–49%. The other four regimens were dominated by pembro‐chemo. For patient with PD‐L1 <1%, nivo‐ipi versus chemotherapy resulted in ICERs of $122,691 per life year and $161,277 per QALY, and the ICERs of nivo‐ipi‐chemo versus nivo‐ipi were $567,261 per life year and $881,975 per QALY. The other regimens were dominated strategies.

**TABLE 2:** Base‐case results

Strategy	Cost (USD)	Life years	QALYs	ICER (USD/life year)	ICER (USD/QALY)
All patients					
Chemotherapy	139,820	1.86	1.39	Reference	Reference
Nivo‐ipi	278,126	2.65	2.02	Weakly dominated	Weakly dominated
Atezo‐chemo	282,282	2.48	1.89	Strongly dominated	Strongly dominated
Nivo‐ipi‐chemo	322,647	2.97	2.24	Weakly dominated	Weakly dominated
Pembro‐chemo	363,468	3.44	2.61	141,790	183,299
Atezo‐beva‐chemo	469,555	3.07	2.39	Strongly dominated	Strongly dominated
Patients with PD‐L1 <1%					
Chemotherapy	116,112	1.54	1.15	Reference	Reference
Atezo‐chemo	226,251	2.00	1.51	Weakly dominated	Weakly dominated
Atezo‐beva‐chemo	231,254	2.09	1.57	Weakly dominated	Weakly dominated
Pembro‐chemo	248,009	2.39	1.79	Weakly dominated	Weakly dominated
Nivo‐ipi	264,556	2.75	2.07	122,691	161,277
Nivo‐ipi‐chemo	312,575	2.84	2.13	567,261	881,975
Patients with PD‐L1 of 1%–49%^a^					
Chemotherapy	142,188	1.72	1.30	Reference	Reference
Pembrolizumab	183,856	1.90	1.47	Weakly dominated	Weakly dominated
Atezo‐chemo	276,079	1.97	1.51	Weakly dominated	Weakly dominated
Nivo‐ipi	290,386	1.90	1.48	Strongly dominated	Strongly dominated
Nivo‐ipi‐chemo	345,346	2.77	2.10	Weakly dominated	Weakly dominated
Pembro‐chemo	360,991	3.02	2.30	168,878	218,159
Patients with PD‐L1 ≥50%					
Chemotherapy	151,703	2.10	1.57	Reference	Reference
Pembrolizumab	228,390	3.12	2.37	74,908	96,189
Nivo‐ipi	321,222	3.19	2.47	Weakly dominated	Weakly dominated
Nivo‐ipi‐chemo	343,656	3.38	2.57	Weakly dominated	Weakly dominated
Atezo‐chemo	401,628	3.68	2.83	Weakly dominated	Weakly dominated
Pembro‐chemo	558,990	5.20	4.03	159,541	198,913

*Note*: Strongly dominated strategies are the ones that have higher costs and fewer QALYs than alternative strategies. Weakly dominated strategies are the ones that are less efficient in terms of incremental costs per QALY as compared with alternative strategies.

Abbreviations: ICER, incremental cost‐effectiveness ratio; PD‐L1, programmed death‐ligand 1; QALY, quality‐adjusted life year; USD, US dollars.

^a^ Survival of chemotherapy group based on the KEYNOTE‐189 trial.

### 3.2 Sensitivity analyses

**FIGURE 2:**
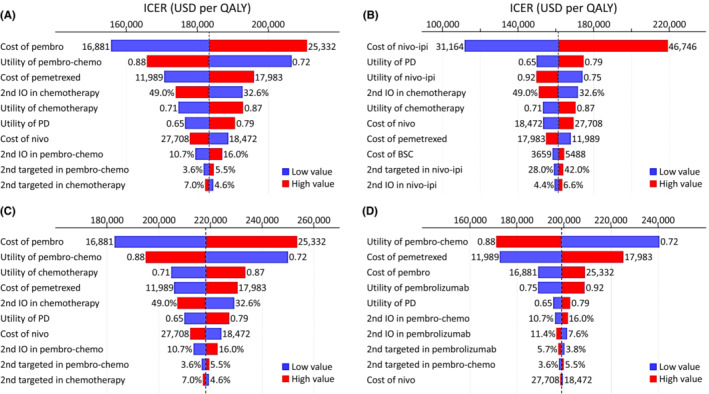
Tornado diagrams for (A) pembro‐chemo versus chemotherapy of all patients; (B) nivo‐ipi versus chemotherapy of patients with PD‐L1 <1%; (C) pembro‐chemo versus chemotherapy of patients with PD‐L1 of 1%–49%; and (D) pembro‐chemo versus pembrolizumab of patients with PD‐L1 ≥50%. The dash lines represent the base‐case ICERs. BSC, best supportive care; ICER, incremental cost‐effectiveness ratio; IO, immunotherapy; PD, progressive disease; PD‐L1, programmed‐death ligand 1; QALY, quality‐adjusted life year; USD, US dollars.

Given the lower and upper bonds of the 95% confidence intervals of hazard ratios, monotherapy with pembrolizumab remained the cost‐effective strategy for patients with PD‐L1 ≥50% (Table 3). Nivo‐ipi and pembro‐chemo continued to be the preferred regimens for patients with PD‐L1 <1% and all patients, respectively. One‐way deterministic sensitivity analyses (Figure 2) show that irrespective of PD‐L1 expression levels, costs of immunotherapies and utility values of immunotherapies were the major determinants of ICER values. Nivo‐ipi would become cost‐effective for patients with PD‐L1 <1% if its cost was decreased to $37,241 (Figure 2B). Varying costs of pemetrexed between 80% and 120% of the baseline value also greatly changed the ICER results for all patients, patients with PD‐L1 of 1%–49%, and patients with PD‐L1 ≥50%.

**TABLE 3:** Sensitivity analyses using the lower and upper bonds of the 95% CIs of hazard ratios for survival estimates

Strategy	Analysis using the lower bonds of 95% CIs			Analysis using the upper bonds of 95% CIs		
	Cost (USD)	QALYs	ICER (USD/QALY)	Cost (USD)	QALYs	ICER (USD/QALY)
All patients						
Chemotherapy	139,820	1.39	Reference	139,820	1.39	Reference
Nivo‐ipi	300,181	2.29	Weakly dominated	256,882	1.76	Weakly dominated
Atezo‐chemo	304,607	2.12	Strongly dominated	259,434	1.66	Strongly dominated
Nivo‐ipi‐chemo	356,550	2.75	Weakly dominated	293,928	1.89	Weakly dominated
Pembro‐chemo	400,606	3.06	156,397	325,777	2.23	222,986
Atezo‐beva‐chemo	571,132	3.07	34,073,405	369,717	1.76	Strongly dominated
Patients with PD‐L1 <1%						
Chemotherapy	116,112	1.15	Reference	116,112	1.15	Reference
Atezo‐chemo	253,329	1.86	Weakly dominated	—	—	—
Atezo‐beva‐chemo	286,321	2.33	Weakly dominated	183,664	1.05	Strongly dominated
Atezo‐chemo	—	—	—	204,411	1.26	Weakly dominated
Pembro‐chemo	290,991	2.35	Weakly dominated	212,568	1.37	Weakly dominated
Nivo‐ipi	304,013	2.64	126,449	226,764	1.60	248,227
Nivo‐ipi‐chemo	367,152	2.92	220,410	261,601	1.51	Strongly dominated
Patients with PD‐L1 of 1%–49%^a^						
Chemotherapy	142,188	1.30	Reference	142,188	1.30	Reference
Pembrolizumab	204,078	1.70	153,428	165,469	1.25	Strongly dominated
Atezo‐chemo	312,441	1.86	Weakly dominated	246,395	1.26	Strongly dominated
Nivo‐ipi	317,569	1.78	Strongly dominated	263,351	1.22	Strongly dominated
Nivo‐ipi‐chemo	402,726	2.80	180,954	289,142	1.58	Weakly dominated
Pembro‐chemo	422,433	2.90	200,243	308,460	1.83	312,540
Patients with PD‐L1 ≥50%						
Chemotherapy	151,703	1.57	Reference	151,703	1.57	Reference
Pembrolizumab	259,449	2.85	84,298	200,043	1.97	120,847
Nivo‐ipi	358,816	3.08	Weakly dominated	—	—	—
Nivo‐ipi‐chemo	415,837	3.67	Weakly dominated	273,607	1.76	Strongly dominated
Nivo‐ipi	—	—	—	282,498	1.96	Strongly dominated
Atezo‐chemo	503,519	3.80	Weakly dominated	316,958	2.06	Weakly dominated
Pembro‐chemo	666,276	5.04	185,542	446,020	3.02	233,552

*Note*: Strongly dominated strategies are the ones that have higher costs and fewer QALYs than alternative strategies. Weakly dominated strategies are the ones that are less efficient in terms of incremental costs per QALY as compared with alternative strategies.

Abbreviations: CI, confidence interval; ICER, incremental cost‐effectiveness ratio; PD‐L1, programmed death‐ligand 1; QALY, quality‐adjusted life year; USD, US dollars.

^a^ Survival of chemotherapy group based on the KEYNOTE‐189 trial.

**FIGURE 3:**
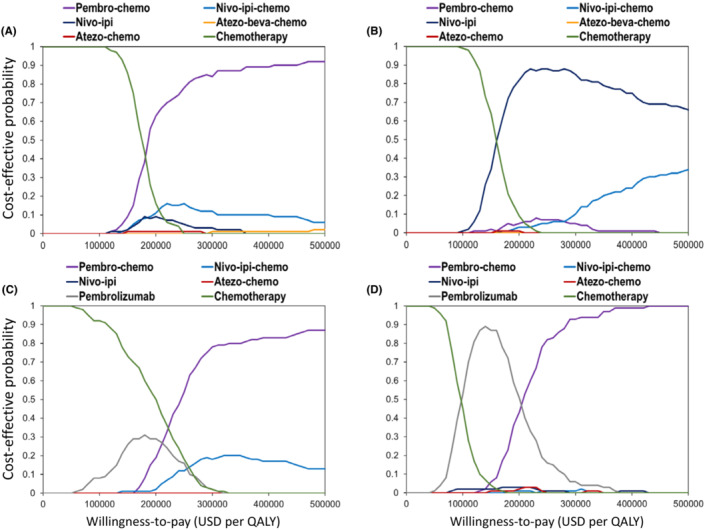
Acceptability curves for (A) all patients; (B) patients with PD‐L1 <1%; (C) patients with PD‐L1 of 1%–49%; and (D) patients with PD‐L1 ≥50%. PD‐L1, programmed‐death ligand 1; QALY, quality‐adjusted life year; USD, US dollars.

Cost‐effectiveness acceptability curves of all patients show that pembro‐chemo had a 9% probability being cost‐effective at the WTP threshold of $150,000 per QALY (Figure 3). The probability for nivo‐ipi became 34% in patients with PD‐L1 <1%. For patients with PD‐L1 of 1%–49%, pembro‐chemo had a 1% probability being cost‐effective at this WTP threshold. The probability was 87% when it applied to pembrolizumab in patients with PD‐L1 ≥50%.

The OS curves, costs, and QALYs of base‐case results and results using trial outcomes of immunotherapy combinations appeared to be similar in all patients, patients with PD‐L1 <1%, and patients with PD‐L1 ≥50% (see Figure S3 and Table S3). However, the survival benefit and QALY gained of the base case were higher than those using trial outcomes of immunotherapy combinations in patients with PD‐L1 of 1%–49%, leading to a lower ICER value.

## 4 DISCUSSION

This cost‐effectiveness analysis (CEA) provides a good opportunity for clinicians to consider efficacy, safety, patients' preferences, and costs when selecting the first‐line immunotherapies. Although CEAs have been performed for individual immunotherapy regimen,^16^, ^17^, ^18^, ^19^, ^20^, ^21^, ^22^, ^23^, ^24^, ^25^, ^26^, ^27^, ^28^, ^29^, ^30^ to the best of our knowledge, there has been no study comparing the cost‐effectiveness across six guideline‐recommended regimens. We stratified patients by PD‐L1 expression levels (<1%, 1%–49%, and ≥50%), which are commonly used in clinical practice. In addition, we well calibrated the PFS and OS curves and weighted the hazard ratios from a network meta‐analysis for transitional probabilities,^31^ our simulation model could accurately estimate the effectiveness. Based on the trial data, we also explicitly modeled the health utility values, adverse events, and subsequent treatments. The results showed that for patients with PD‐L1 ≥50%, monotherapy with pembrolizumab was more likely to be cost‐effective as compared to other regimens; whereas for patients with PD‐L1 of 1%–49% and <1%, pembro‐chemo and nivo‐ipi were the preferred immunotherapy strategies, respectively. This study may help thoracic oncologists move toward value‐based practice while treating patients with advanced NSCLC whose tumors lack of actionable gene alterations.

Pembro‐chemo, nivo‐ipi‐chemo, atezo‐beva‐chemo, and atezo‐chemo shared similar profiles of health utility and safety. However, anti‐PD‐L1 combinations (atezo‐beva‐chemo and atezo‐chemo) were dominated by anti‐PD‐1 combinations (pembro‐chemo and nivo‐ipi‐chemo). This finding is consistent with previous CEA results which failed to prove atezolizumab combinations to be cost‐effective.^28^, ^29^, ^30^ A dominated strategy of anti‐PD‐L1 combination was mainly explained by its less favorable OS as compared to an anti‐PD‐1 combination. Exhibiting an unfavorable OS of anti‐PD‐L1 combinations could be attributable to the fact that anti‐PD‐L1 only inhibits PD‐L1, whereas anti‐PD‐1 inhibits the binding of PD‐1 to both PD‐L1 and PD‐L2, which in turn blocks the immune escape more comprehensively.^47^ Among anti‐PD‐1 combinations, nivo‐ipi was more likely to be cost‐effective for patients with PD‐L1 <1%, and pembro‐chemo was the preferred regimen for patients with PD‐L1 ≥1%. These findings were corroborated by an observation that PD‐L1 expression levels might not be a reliable biomarker in judging the effectiveness of immunotherapy combinations including anti‐CTLA‐4 therapy.^48^

As expected, tornado diagram reveals that the cost and utility value of each immunotherapy were the major determinants of ICER. In this figure, we also recognized that cost of pemetrexed is a major determinant of pembro‐chemo cost‐effectiveness. Maintenance therapy of pemetrexed was administered in pembro‐chemo,^7^ atezo‐chemo,^11^ and their chemotherapy groups. However, it was not applied to nivo‐ipi‐chemo and nivo‐ipi,^13^, ^14^ and was optional for their chemotherapy groups. A superior survival benefit of pembro‐chemo as compared to nivo‐ipi or nivo‐ipi‐chemo might result from the effect of pemetrexed maintenance. If we did not consider the cost of pemetrexed maintenance in each group, pembro‐chemo would be a cost‐effective strategy for all patients and patients with PD‐L1 ≥50% (see Table S4).

We acknowledge that the WTP threshold of $150,000 per QALY might be a low estimate given the increase in healthcare spending. If we used a threshold of $200,000 per QALY (the “three times gross domestic product per capita cost‐effectiveness threshold” proposed by the World Health Organization),^49^ pembro‐chemo would become cost‐effective for patients with PD‐L1 ≥50%. Nivo‐ipi would be a cost‐effective strategy for patients with PD‐L1 <1%. Nivo‐ipi‐chemo and atezo‐chemo, however, would remain not cost‐effective regardless of PD‐L1 levels and should be discouraged.

Our target population were NSCLC patients whose tumors lack of actionable gene alterations. Most of these patients were current or former smokers who might be eligible for low‐dose chest tomography (LDCT) screening in the early beginning of the disease. From the perspective of US health care sector, the ICERs for immunotherapy combinations were much higher than those estimated for LDCT screening.^50^, ^51^ Although the comparison groups were different, our results emphasize a potential need for the shift to detecting early‐stage lung cancer among high‐risk smokers.

Several limitations must be acknowledged in our study. First, because of a lack of survival curves for patients with PD‐L1 of 1%–49% in the CheckMate 227 trial,^13^ we used the PFS and OS curves of KEYNOTE‐189 chemotherapy group for modeling.^7^ The hazard ratios of pembro‐chemo versus chemotherapy in the network meta‐analysis^31^ were lower than those in the KEYNOTE‐189 trial,^7^ leading to a lower ICER estimate. Nevertheless, our results still indicated that pembro‐chemo was not a cost‐effective strategy in patients with PD‐L1 of 1%–49%. We also acknowledged the limitation that we assumed constant hazard ratios, which is often incorrect. However, these hazard ratios, derived from network meta‐analysis,^31^ represent the best evidence while comparing multiple immunotherapy regimens. Furthermore, the modeled and observed survival curves were similar, indicating that our assumption/model is still appropriate. Second, we only compared the cost‐effectiveness of six guideline‐recommended immunotherapies. Atezolizumab and cemiplimab‐rwlc monotherapies were also recommended as the front‐line immunotherapy for patients with PD‐L1 ≥50%,^15^ but we did not simultaneously compare their cost‐effectiveness. Besides, the hazard ratios of pembrolizumab versus chemotherapy and the adverse event rates were directly derived from the KEYNOTE‐042 study^5^ without cross‐trial adjustment. Investigations using meta‐analysis across immunotherapy monotherapy and immunotherapy combinations merit future research. Third, based on the CheckMate 227 trial,^13^ we assumed 28.0% of tumors was squamous in histology when simulated patients entered the model. Atezo‐beva‐chemo should not be administered to patients with squamous NSCLC.^15^ However, we assigned paclitaxel plus carboplatin as the chemotherapy regimen for both squamous and non‐squamous NSCLC in this group of patients, the validity of our results would not be threatened. Fourth, we applied a fixed utility value for each immunotherapy, which might not capture the decrements of quality of life resulting from aging or co‐morbidities. Nevertheless, patients with advanced NSCLC usually experience a short life expectancy, the QALYs of patients should not be overestimated too much.

In conclusion, from the perspective of US health care sector, pembrolizumab, pembro‐chemo, and nivo‐ipi are the preferred first‐line regimens for patients with PD‐L1 ≥50%, 1%–49%, and <1%, respectively. Atezo‐beva‐chemo and atezo‐chemo are unlikely to be cost‐effective regardless of PD‐L1 expression levels.

## AUTHOR CONTRIBUTIONS

**Szu‐Chun Yang:** Conceptualization, collection and assembly of data, formal analysis, funding acquisition, project administration, visualization, writing–original draft, writing–review/editing. **Huang‐Tz Ou:** Collection and assembly of data, data curation, writing–review/editing. **Wu‐Chou Su:** Conceptualization, resources, writing–review/editing. **Shi‐Yi Wang:** Conceptualization, methodology, supervision, writing–review/editing.

## FUNDING INFORMATION

The work was supported by the Ministry of Science and Technology (110‐2314‐B‐006‐100‐MY2) and National Cheng Kung University Hospital (NCKUH‐11203001). The funding organization had no role in the design and conduct of the study; collection, management, analysis, and interpretation of the data; preparation, review, or approval of the manuscript; and decision to submit the manuscript for publication.

## CONFLICT OF INTEREST

Dr. Yang reports grants from the Ministry of Science and Technology and National Cheng Kung University Hospital during the conduct of the study. No other disclosures were reported.

## ETHICS APPROVAL STATEMENT

The study was exempt from gaining individual consent, and no ethical approval was required for the study, as it involved the analysis of previously published data.

## Supporting information


Data S1

## Data Availability

The data that support the findings of this study are available from the corresponding author upon reasonable request.
