# Supplementary material for: Cost‐effectiveness of first‐line immunotherapies for advanced non‐small cell lung cancer
Source: Cancer Med. 2023 Jan 18;12(7):8838–50. doi: 10.1002/cam4.5632 (PMC10134257; doi:10.1002/cam4.5632)
Supplement: Supplementary file 1 — Data S1 [file CAM4-12-8838-s001.doc]

Supporting information

**Cost-effectiveness of first-line immunotherapies for advanced non-small cell lung cancer**

**Figure S1.** Model structure. Patients were treated with one of the seven first-line therapies: pembrolizumab plus chemotherapy (pembro-chemo), nivolumab plus ipilimumab (nivo-ipi), nivolumab and ipilimumab plus chemotherapy (nivo-ipi-chemo), atezolizumab plus chemotherapy (atezo-chemo), atezolizumab and bevacizumab plus chemotherapy (atezo-beva-chemo), single-agent pembrolizumab, and chemotherapy alone. NSCLC, non-small cell lung cancer.


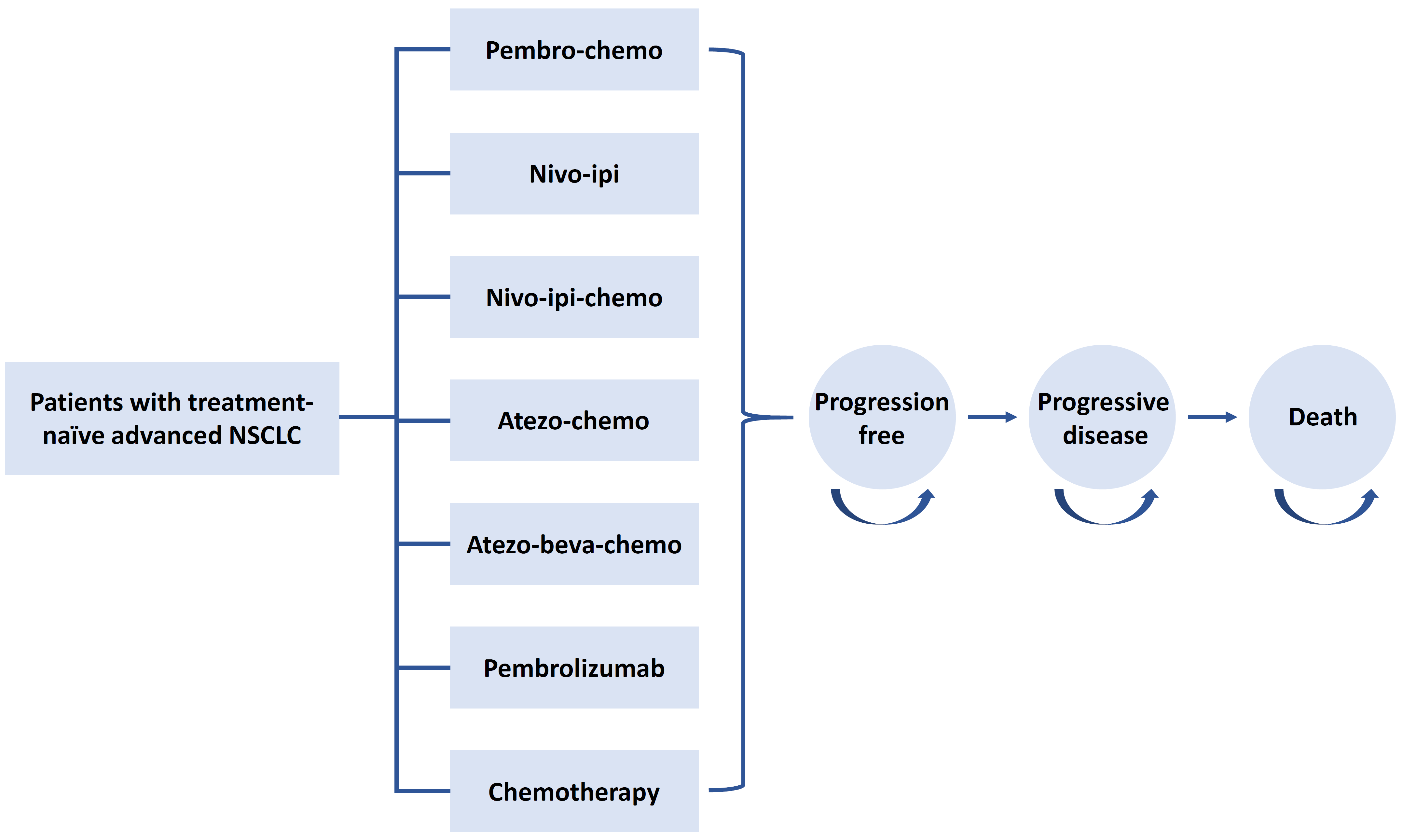


**Figure S2.** Trial and modeled PFS and OS curves for (A) all patients; (B) patients with PD-L1 < 1%; (C) patients with PD-L1 of 1-49%; and (D) patients with PD-L1 ≥ 50%. We extrapolated (dash line) the survival to lifetime for analysis. PD-L1, programmed-death ligand 1; PFS, progression-free survival; OS, overall survival.


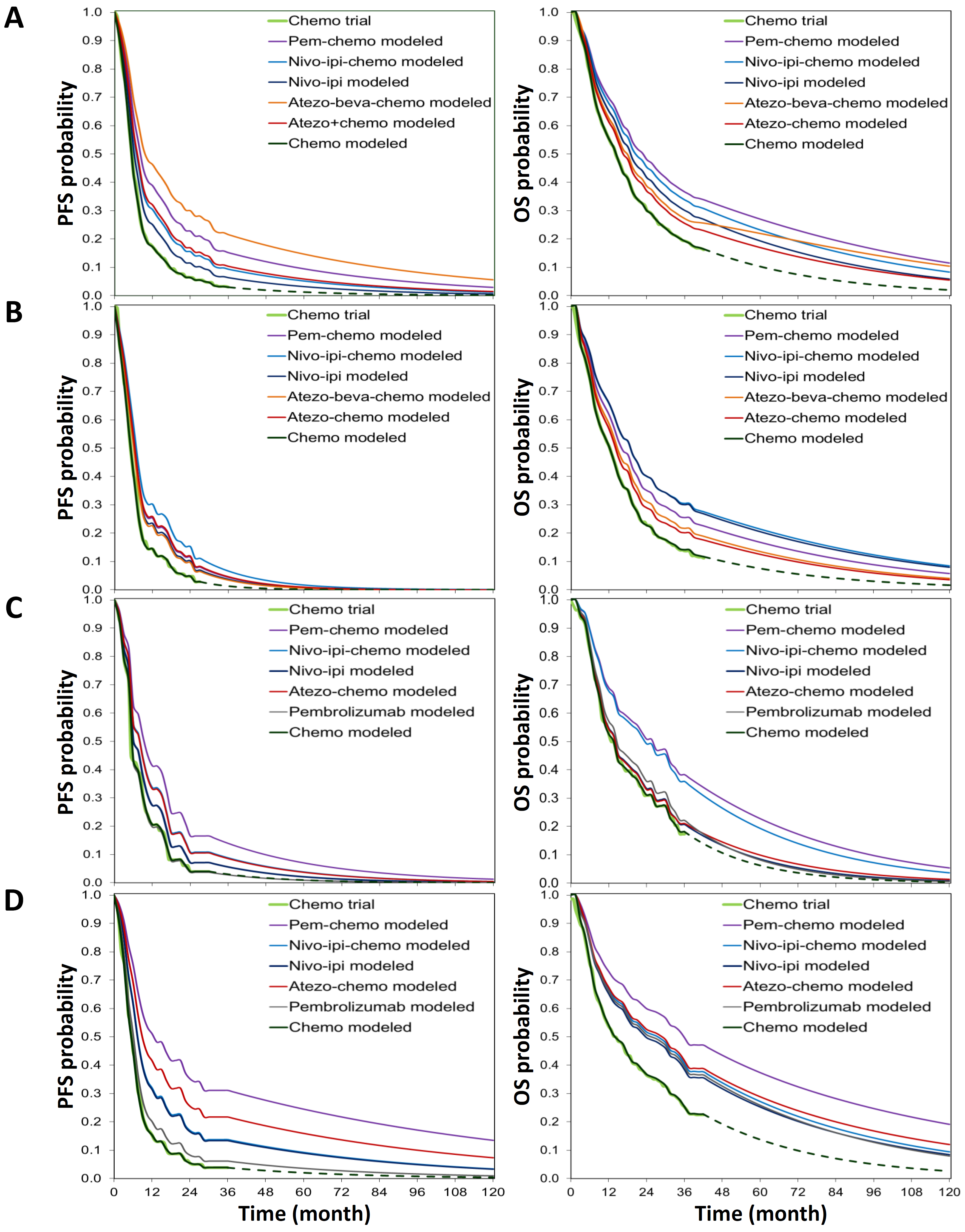


**Figure S3.** Trial and modeled overall survival (OS) curves of immunotherapy combinations for (A) all patients; (B) patients with PD-L1 < 1%; (C) patients with PD-L1 of 1-49%; and (D) patients with PD-L1 ≥ 50%. PD-L1, programmed-death ligand 1.


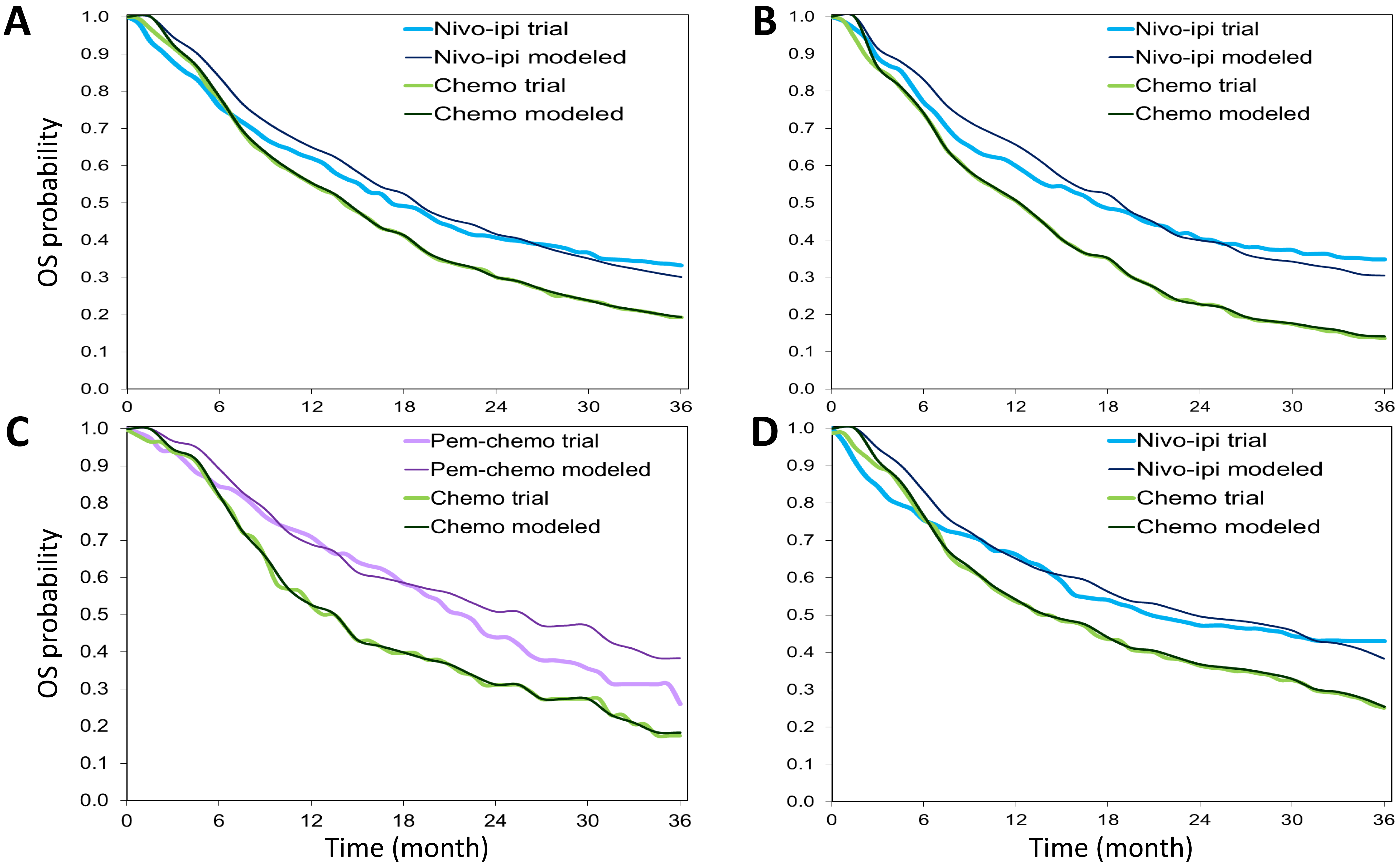


| **Table S1.** Doses and costs of drugs | | | |
| --- | --- | --- | --- |
| Drug^a^ | Dose | Unit price | Cost per 6 weeks |
| Pembrolizumab | 200 mg every 3 weeks up to 2 years | $52.754 per 1 mg | $21,102 |
| Nivolumab | 360 mg every 3 weeks up to 2 years | $28.863 per 1 mg | $23,090 |
| Ipilimumab | 1 mg/kg * 70 kg every 6 weeks up to 2 years | $158.648 per 1 mg | $15,865 |
| Atezolizumab | 1200 mg every 3 weeks up to 2 years | $7.975 per 1 mg | $19,140 |
| Bevacizumab | 15 mg/kg * 70 kg every 3 weeks | $7.041 per 1 mg | $15,491 |
| Pemetrexed | 500 mg/m^2^ * 1.84 m^2^ every 3 weeks | $7.493 per 1 mg | $14,986 |
| Carboplatin | 490 mg (AUC: 5 mg/ml/min) every 3 weeks | $5.286 per 100 mg | $63 |
| Paclitaxel | 200 mg/m^2^ * 1.84 m^2^ every 3 weeks | $0.131 per 1 mg | $105 |
| Docetaxel | 75 mg/m^2^ *1.84 m^2^ every 3 weeks up to 12 weeks | $0.479 per 1 mg | $134 |
| Erlotinib | 150 mg per day | $313.024 per 150 mg | $13,147 |
| Abbreviations: AUC, area under the concentration-time curve.  ^a^ Pembrolizumab: 100 mg/vial; nivolumab: 100 mg/vial; ipilimumab: 50 mg/vial; atezolizumab: 1200 mg/vial; bevacizumab: 100 mg/vial; pemetrexed: 500 mg/vial; carboplatin: 150 mg/vial; paclitaxel: 100 mg/vial; docetaxel: 20 mg/vial. | | | |

| **Table S2.** Parameter values for adverse events (AEs) | | | | |
| --- | --- | --- | --- | --- |
| Parameter | Value | Range | Distribution | Source |
| Incidence of AE, Pembro-chemo | | | | |
| Fatigue | 0.37 | 0.30-0.44 | Beta (277,471) | Network meta-analysis(1) |
| Nausea | 0.49 | 0.39-0.59 | Beta (367,381) | Network meta-analysis(1) |
| Anemia | 0.48 | 0.38-0.58 | Beta (359,389) | Network meta-analysis(1) |
| Vomiting | 0.22 | 0.18-0.26 | Beta (165,583) | Network meta-analysis(1) |
| Diarrhea | 0.31 | 0.25-0.37 | Beta (232,516) | Network meta-analysis(1) |
| Anorexia | 0.27 | 0.22-0.32 | Beta (202,546) | Network meta-analysis(1) |
| Constipation | 0.30 | 0.24-0.36 | Beta (224,524) | Network meta-analysis(1) |
| Neutropenia | 0.30 | 0.24-0.36 | Beta (224,524) | Network meta-analysis(1) |
| Hypothyroidism | 0.09 | 0.07-0.11 | Beta (67,681) | Network meta-analysis(1) |
| Hyperthyroidism | 0.06 | 0.05-0.07 | Beta (45,703) | Network meta-analysis(1) |
| Rash | 0.20 | 0.16-0.24 | Beta (150,598) | Network meta-analysis(1) |
| Pruritus | 0.08 | 0.06-0.10 | Beta (60,688) | Network meta-analysis(1) |
| Pneumonitis | 0.06 | 0.05-0.07 | Beta (45,703) | Network meta-analysis(1) |
| Colitis | 0.03 | 0.02-0.04 | Beta (22,726) | Network meta-analysis(1) |
| Incidence of AE, Nivo-ipi | | | | |
| Fatigue | 0.14 | 0.11-0.17 | Beta (82,501) | Network meta-analysis(1) |
| Nausea | 0.10 | 0.08-0.12 | Beta (58,525) | Network meta-analysis(1) |
| Anemia | 0.04 | 0.03-0.05 | Beta (23,560) | Network meta-analysis(1) |
| Vomiting | 0.05 | 0.04-0.06 | Beta (29,554) | Network meta-analysis(1) |
| Diarrhea | 0.17 | 0.14-0.20 | Beta (99,484) | Network meta-analysis(1) |
| Anorexia | 0.13 | 0.10-0.16 | Beta (76,507) | Network meta-analysis(1) |
| Constipation | 0.05 | 0.04-0.06 | Beta (29,554) | Network meta-analysis(1) |
| Neutropenia | 0.01 | 0.01-0.01 | Beta (6,577) | Network meta-analysis(1) |
| Rash | 0.17 | 0.14-0.20 | Beta (99,484) | Network meta-analysis(1) |
| Pruritus | 0.14 | 0.11-0.17 | Beta (82,501) | Network meta-analysis(1) |
| Pneumonitis | 0.01 | 0.01-0.01 | Beta (4,579) | Network meta-analysis(1) |
| Incidence of AE, Nivo-ipi-chemo | | | | |
| Fatigue | 0.25 | 0.20-0.30 | Beta (90,271) | Network meta-analysis(1) |
| Nausea | 0.39 | 0.31-0.47 | Beta (141,220) | Network meta-analysis(1) |
| Anemia | 0.41 | 0.33-0.49 | Beta (148,213) | Network meta-analysis(1) |
| Vomiting | 0.15 | 0.12-0.18 | Beta (54,307) | Network meta-analysis(1) |
| Diarrhea | 0.10 | 0.08-0.12 | Beta (36,325) | Network meta-analysis(1) |
| Anorexia | 0.23 | 0.18-0.28 | Beta (83,278) | Network meta-analysis(1) |
| Constipation | 0.22 | 0.18-0.26 | Beta (79,282) | Network meta-analysis(1) |
| Neutropenia | 0.16 | 0.13-0.19 | Beta (58,303) | Network meta-analysis(1) |
| Hypothyroidism | 0.15 | 0.12-0.18 | Beta (54,307) | Network meta-analysis(1) |
| Hyperthyroidism | 0.08 | 0.06-0.10 | Beta (29,332) | Network meta-analysis(1) |
| Rash | 0.15 | 0.12-0.18 | Beta (54,307) | Network meta-analysis(1) |
| Pruritus | 0.09 | 0.07-0.11 | Beta (32,329) | Network meta-analysis(1) |
| Pneumonitis | 0.02 | 0.02-0.02 | Beta (7,354) | Network meta-analysis(1) |
| Colitis | 0.01 | 0.01-0.01 | Beta (4,357) | Network meta-analysis(1) |
| Incidence of AE, Atezo-chemo | | | | |
| Fatigue | 0.33 | 0.26-0.40 | Beta (474,962) | Network meta-analysis(1) |
| Nausea | 0.37 | 0.30-0.44 | Beta (531,905) | Network meta-analysis(1) |
| Anemia | 0.48 | 0.38-0.58 | Beta (689,747) | Network meta-analysis(1) |
| Vomiting | 0.18 | 0.14-0.22 | Beta (258,1178) | Network meta-analysis(1) |
| Diarrhea | 0.29 | 0.23-0.35 | Beta (416,1020) | Network meta-analysis(1) |
| Anorexia | 0.24 | 0.19-0.29 | Beta (345,1091) | Network meta-analysis(1) |
| Constipation | 0.23 | 0.18-0.28 | Beta (330,1106) | Network meta-analysis(1) |
| Neutropenia | 0.26 | 0.21-0.31 | Beta (373,1063) | Network meta-analysis(1) |
| Hypothyroidism | 0.11 | 0.09-0.13 | Beta (158,1278) | Network meta-analysis(1) |
| Hyperthyroidism | 0.04 | 0.03-0.05 | Beta (57,1379) | Network meta-analysis(1) |
| Rash | 0.11 | 0.09-0.13 | Beta (158,1278) | Network meta-analysis(1) |
| Pruritus | 0.05 | 0.04-0.06 | Beta (72,1364) | Network meta-analysis(1) |
| Pneumonitis | 0.08 | 0.06-0.10 | Beta (115,1321) | Network meta-analysis(1) |
| Colitis | 0.02 | 0.02-0.02 | Beta (29,1407) | Network meta-analysis(1) |
| Incidence of AE, Atezo-beva-chemo | | | | |
| Fatigue | 0.26 | 0.21-0.31 | Beta (93,263) | Network meta-analysis(1) |
| Nausea | 0.34 | 0.27-0.41 | Beta (121,235) | Network meta-analysis(1) |
| Anemia | 0.24 | 0.19-0.29 | Beta (85,271) | Network meta-analysis(1) |
| Vomiting | 0.14 | 0.11-0.17 | Beta (50,306) | Network meta-analysis(1) |
| Diarrhea | 0.21 | 0.17-0.25 | Beta (75,281) | Network meta-analysis(1) |
| Anorexia | 0.22 | 0.18-0.26 | Beta (78,278) | Network meta-analysis(1) |
| Constipation | 0.17 | 0.14-0.20 | Beta (61,295) | Network meta-analysis(1) |
| Neutropenia | 0.12 | 0.10-0.14 | Beta (43,313) | Network meta-analysis(1) |
| Hypothyroidism | 0.13 | 0.10-0.16 | Beta (46,310) | Network meta-analysis(1) |
| Hyperthyroidism | 0.04 | 0.03-0.05 | Beta (14,342) | Network meta-analysis(1) |
| Rash | 0.13 | 0.10-0.16 | Beta (46,310) | Network meta-analysis(1) |
| Pneumonitis | 0.03 | 0.02-0.04 | Beta (11,345) | Network meta-analysis(1) |
| Colitis | 0.02 | 0.02-0.02 | Beta (7,349) | Network meta-analysis(1) |
| Incidence of AE, Pembrolizumab | | | | |
| Fatigue | 0.08 | 0.06-1.10 | Beta(51,586) | KEYNOTE-042 trial(2) |
| Nausea | 0.05 | 0.04-0.06 | Beta(31,606) | KEYNOTE-042 trial(2) |
| Anemia | 0.06 | 0.04-0.07 | Beta(35,602) | KEYNOTE-042 trial(2) |
| Vomiting | 0.02 | 0.02-0.03 | Beta(15,622) | KEYNOTE-042 trial(2) |
| Anorexia | 0.06 | 0.05-0.08 | Beta(40,597) | KEYNOTE-042 trial(2) |
| Constipation | 0.01 | 0.01-0.02 | Beta(8,629) | KEYNOTE-042 trial(2) |
| Neutropenia | 0.01 | 0.01-0.01 | Beta(5,632) | KEYNOTE-042 trial(2) |
| Hypothyroidism | 0.11 | 0.09-0.13 | Beta(69,568) | KEYNOTE-042 trial(2) |
| Hyperthyroidism | 0.06 | 0.05-0.07 | Beta(38,599) | KEYNOTE-042 trial(2) |
| Rash | 0.02 | 0.02-0.03 | Beta(14,623) | KEYNOTE-042 trial(2) |
| Pneumonitis | 0.08 | 0.07-0.10 | Beta(52,585) | KEYNOTE-042 trial(2) |
| Colitis | 0.01 | 0.01-0.02 | Beta(8,629) | KEYNOTE-042 trial(2) |
| Incidence of AE, Chemotherapy | | | | |
| Fatigue | 0.19 | 0.15-0.23 | Beta (446,1899) | CheckMate 227 trial(3) |
| Nausea | 0.36 | 0.29-0.43 | Beta (844,1501) | CheckMate 227 trial(3) |
| Anemia | 0.33 | 0.26-0.40 | Beta (774,1571) | CheckMate 227 trial(3) |
| Vomiting | 0.14 | 0.11-0.16 | Beta (317,2028) | CheckMate 227 trial(3) |
| Diarrhea | 0.10 | 0.08-0.12 | Beta (235,2111) | CheckMate 227 trial(3) |
| Anorexia | 0.20 | 0.16-0.24 | Beta (469,1876) | CheckMate 227 trial(3) |
| Constipation | 0.15 | 0.12-0.18 | Beta (350,1995) | CheckMate 227 trial(3) |
| Neutropenia | 0.17 | 0.14-0.20 | Beta (399,1946) | CheckMate 227 trial(3) |
| Rash | 0.05 | 0.04-0.06 | Beta (117,2228) | CheckMate 227 trial(3) |
| Pruritus | 0.01 | 0.01-0.01 | Beta (25,2320) | CheckMate 227 trial(3) |
| Cost for AE, USD | | | | |
| Fatigue | 733 | 586-880 | Gamma (100,7.33) | Medicare analysis(4) |
| Nausea | 1965 | 1572-2358 | Gamma (100,19.65) | Claims database analysis(5) |
| Anemia | 4353 | 3482-5224 | Gamma (100,43.53) | Claims database analysis(5) |
| Vomiting | 895 | 716-1074 | Gamma (100,8.95) | Claims database analysis(5) |
| Diarrhea | 3265 | 2612-3918 | Gamma (100,32.65) | Claims database analysis(5) |
| Anorexia | 3700 | 2960-4440 | Gamma (100,37.00) | Claims database analysis(5) |
| Constipation | 2591 | 2073-3109 | Gamma (100,25.91) | Claims database analysis(5) |
| Neutropenia | 5321 | 4257-6385 | Gamma (100,53.21) | Claims database analysis(5) |
| Hypothyroidism | 2255 | 1804-2706 | Gamma (100,22.55) | Claims database analysis(5) |
| Hyperthyroidism | 2255 | 1804-2706 | Gamma (100,22.55) | Claims database analysis(5) |
| Rash | 940 | 752-1128 | Gamma (100,9.40) | Claims database analysis(5) |
| Pruritus | 1184 | 947-1421 | Gamma (100,11.84) | Claims database analysis(5) |
| Pneumonitis | 9941 | 7953-11,929 | Gamma (100,99.41) | Claims database analysis(5) |
| Colitis | 6079 | 4863-7295 | Gamma (100,60.79) | Claims database analysis(5) |

**References:**

1. Liu L, Bai H, Wang C, Seery S, Wang Z, Duan J, et al. Efficacy and safety of first-line immunotherapy combinations for advanced NSCLC: a systematic review and network meta-analysis. J Thorac Oncol. 2021;16(7):1099-117.

2. de Castro G, Jr., Kudaba I, Wu YL, Lopes G, Kowalski DM, Turna HZ, et al. Five-year outcomes with pembrolizumab versus chemotherapy as first-line therapy in patients with non-small-cell lung cancer and programmed death ligand-1 tumor proportion score ≥ 1% in the KEYNOTE-042 study. J Clin Oncol. 2022:JCO2102885. doi: 10.1200/JCO.21.02885. Online ahead of print.

3. Hellmann MD, Paz-Ares L, Bernabe Caro R, Zurawski B, Kim SW, Carcereny Costa E, et al. Nivolumab plus ipilimumab in advanced non-small-cell lung cancer. N Engl J Med. 2019;381(21):2020-31.

4. Bittoni MA, Arunachalam A, Li H, Camacho R, He J, Zhong Y, et al. Real-world treatment patterns, overall survival, and occurrence and costs of adverse events associated with first-line therapies for Medicare patients 65 years and older with advanced non-small-cell lung cancer: a retrospective study. Clin Lung Cancer. 2018;19(5):e629-e45.

5. Wong W, Yim YM, Kim A, Cloutier M, Gauthier-Loiselle M, Gagnon-Sanschagrin P, et al. Assessment of costs associated with adverse events in patients with cancer. PloS One. 2018;13(4):e0196007.

| **Table S3.** Comparison between base-case results and results using trial outcomes of immunotherapy combinations | | | | | | | |
| --- | --- | --- | --- | --- | --- | --- | --- |
|  | Base case modeling outcomes using the HRs in a network meta-analysis | | |  | Results using trial outcomes of immunotherapy combinations | | |
| Strategy | Cost (USD) | QALYs | ICER (USD/QALY) |  | Cost (USD) | QALYs | ICER (USD/QALY) |
| All patients | | | | | | | |
| Chemotherapy | 139,820 | 1.39 | Reference |  | -- | -- | -- |
| Nivo-ipi | 278,126 | 2.02 | 221,486 |  | 249,809 | 1.96 | 193,658 |
| Patients with PD-L1 < 1% | | | | | | | |
| Chemotherapy | 116,112 | 1.15 | Reference |  | -- | -- | -- |
| Nivo-ipi | 264,556 | 2.07 | 161,277 |  | 266,767 | 2.17 | 148,617 |
| Patients with PD-L1 of 1-49%^a^ | | | | | | | |
| Chemotherapy | 142,188 | 1.30 | Reference |  | -- | -- | -- |
| Pembro-chemo | 360,991 | 2.30 | 218,159 |  | 335,771 | 1.84 | 355,477 |
| Patients with PD-L1 ≥ 50% | | | | | | | |
| Chemotherapy | 151,703 | 1.57 | Reference |  | -- | -- | -- |
| Nivo-ipi | 321,222 | 2.47 | 187,843 |  | 292,129 | 2.40 | 169,258 |
| Abbreviations: HR, hazard ratio; ICER, incremental cost-effectiveness ratio; PD-L1, programmed death-ligand 1; QALY, quality-adjusted life year; USD, US dollars.  ^a^ Survival of chemotherapy group based on the KEYNOTE-189 trial. | | | | | | | |

| **Table S4.** Results without considering the cost of pemetrexed maintenance | | | | | |
| --- | --- | --- | --- | --- | --- |
| Strategy | Cost (USD) | Life years | QALYs | ICER  (USD/life year) | ICER  (USD/QALY) |
| All patients | | | | | |
| Chemotherapy | 96,785 | 1.86 | 1.39 | Reference | Reference |
| Atezo-chemo | 196,313 | 2.48 | 1.89 | Weekly dominated | Weekly dominated |
| Pembro-chemo | 245,069 | 3.44 | 2.61 | 94,010 | 121,532 |
| Nivo-ipi | 278,126 | 2.65 | 2.02 | Strongly dominated | Strongly dominated |
| Nivo-ipi-chemo | 322,647 | 2.97 | 2.24 | Strongly dominated | Strongly dominated |
| Atezo-beva-chemo | 469,555 | 3.07 | 2.39 | Strongly dominated | Strongly dominated |
| Patients with PD-L1 < 1% | | | | | |
| Chemotherapy | 84,517 | 1.54 | 1.15 | Reference | Reference |
| Atezo-chemo | 174,277 | 2.00 | 1.51 | Weakly dominated | Weakly dominated |
| Pembro-chemo | 197,133 | 2.39 | 1.79 | 133,218 | 176,773 |
| Atezo-beva-chemo | 231,254 | 2.09 | 1.57 | Strongly dominated | Strongly dominated |
| Nivo-ipi | 264,556 | 2.75 | 2.07 | 184,949 | 237,935 |
| Nivo-ipi-chemo | 312,575 | 2.84 | 2.13 | 567,261 | 881,975 |
| Patients with PD-L1 of 1-49%^a^ | | | | | |
| Chemotherapy | 94,203 | 1.72 | 1.30 | Reference | Reference |
| Pembrolizumab | 158,008 | 1.90 | 1.47 | Weakly dominated | Weakly dominated |
| Atezo-chemo | 198,356 | 1.97 | 1.51 | Weakly dominated | Weakly dominated |
| Pembro-chemo | 253,354 | 3.02 | 2.30 | 122,837 | 158,682 |
| Nivo-ipi | 290,386 | 1.90 | 1.48 | Strongly dominated | Strongly dominated |
| Nivo-ipi-chemo | 345,346 | 2.77 | 2.10 | Strongly dominated | Strongly dominated |
| Patients with PD-L1 ≥ 50% | | | | | |
| Chemotherapy | 103,708 | 2.10 | 1.57 | Reference | Reference |
| Pembrolizumab | 169,613 | 3.12 | 2.37 | Weakly dominated | Weakly dominated |
| Atezo-chemo | 229,285 | 3.68 | 2.83 | Weakly dominated | Weakly dominated |
| Pembro-chemo | 284,560 | 5.20 | 4.03 | 58,416 | 73,538 |
| Nivo-ipi | 321,222 | 3.19 | 2.47 | Strongly dominated | Strongly dominated |
| Nivo-ipi-chemo | 343,656 | 3.38 | 2.57 | Strongly dominated | Strongly dominated |
| Note: Strongly dominated strategies are the ones that have higher costs and fewer QALYs than alternative strategies. Weakly dominated strategies are the ones that are less efficient in terms of incremental costs per QALY as compared with alternative strategies.  Abbreviations: ICER, incremental cost-effectiveness ratio; PD-L1, programmed death-ligand 1; QALY, quality-adjusted life year; USD, US dollars.  ^a^ Survival of chemotherapy group based on the KEYNOTE-189 trial. | | | | | |
